# Retraction Note: miR-1301 inhibits hepatocellular carcinoma cell migration, invasion, and angiogenesis by decreasing Wnt/*β*-catenin signaling through targeting BCL9

**DOI:** 10.1038/s41419-023-05765-5

**Published:** 2023-03-29

**Authors:** Chao Yang, Yonghua Xu, Feng Cheng, Yuanchang Hu, Shikun Yang, Jianhua Rao, Xuehao Wang

**Affiliations:** 1 Department of Liver Surgery/Liver Transplantation Center, The First Affiliated Hospital of Nanjing Medical University, Key Laboratory on Living Donor Liver Transplantation of National Health and Family Planning Commission of China, Nanjing, China; 2 Department of General Surgery, Yancheng City No.1 People’s Hospital/The Fourth Affiliated Hospital of Nantong University, Yancheng, China

Retraction to: *Cell Death and Disease* 10.1038/cddis.2017.356, published online 17 August 2017

The Editors have retracted this article. After publication, concerns were raised regarding overlap in the migration and invasion assay images presented in the figures, as well as the cell lines used in the study. Specifically:

- In Fig. 2E and F, the NC images appear to contain overlap.
- There appear to be a number of overlapping images in Figs. 3A-D and 6E and F.
- L02 and SMMC-7721 cell lines have been reported to be contaminated with HeLa and are therefore unsuitable for liver cancer research.
- HepG2 cells have been reported to be unsuitable as a model of hepatocellular carcinoma.

The authors have checked the original data and stated that the image errors occurred due to incorrect data storage. The Editors and authors therefore no longer have confidence in the presented data.

Chao Yang has stated on behalf of all authors that they agree to this retraction.

